# Prenatal and postnatal manifestations of *WBP11*-related disorder in Chinese patients: expanding the phenotypic and mutational spectrum

**DOI:** 10.1186/s40246-026-00966-3

**Published:** 2026-04-13

**Authors:** Tingbin Ma, Jinyu Liu, Yuqi Wang, Haibo Zhu, Yihong Qin, Ruizhi Liu, Hongtao Yuan, Baoying Ye, Renyi Hua, Shuyuan Li, Hui Xi, Jian Wang, Niu Li

**Affiliations:** 1 International Peace Maternity and Child Health Hospital, Shanghai Jiao Tong University School of Medicine, Shanghai, China; 2 Shanghai Key Laboratory of Embryo Original Diseases, Shanghai, China; 3 Faculty of Medical Laboratory Science, College of Health Science and Technology, Shanghai Jiao Tong University School of Medicine, Shanghai, China; 4 Institute of Birth Defects and Rare Diseases, Shanghai Jiao Tong University School of Medicine, Shanghai, China; 5 Department of Child Health Care, Baoding Maternal and Child Health Care Hospital, Baoding, Hebei China; 6 Center of Reproductive Medicine and Center of Prenatal Diagnosis, First Hospital, Jilin University, Changchun, China; 7 Department of Medical Genetics, NHC Key Laboratory of Birth Defect for Research and Prevention (Hunan Provincial Maternal and Child Health Care Hospital), Changsha, China; 8 Center for Prenatal Diagnosis, Baoding Maternal and Child Health Care Hospital, Baoding, Hebei China

**Keywords:** *WBP11*, VACTERL syndrome, Prenatal diagnosis, Phenotypic heterogeneity, Splicing variant, Incomplete penetrance

## Abstract

**Background:**

Heterozygous pathogenic variants in *WBP11*, a spliceosome-associated gene, have recently been linked to VACTERL syndrome, yet prenatal manifestations and genotype–phenotype correlations remain poorly characterized.

**Methods:**

Genomic DNA was extracted from fetal tissues, amniotic fluid cells, or peripheral blood samples for trio-based whole-exome sequencing (WES) to identify potential genetic etiologies. The structural stability of the WBP11 protein associated with missense variants was characterized using 3D protein structural modeling. The impact of splicing variant was further evaluated through TA cloning coupled with Sanger sequencing.

**Results:**

Here, we report the first case series of *WBP11*-related disorder in Chinese patients, comprising four fetuses and one postnatal child from five unrelated families. Whole-exome sequencing identified five previously unreported heterozygous *WBP11* variants (NM_016312.3), including three nonsense (p.Arg55*, p.Lys83*, p.Ser279*), one canonical splice-site (c.1310-1G > A), and one missense (p.Arg91Cys). Three occurred de novo, while two were paternally inherited from clinically unaffected or mildly affected carriers, supporting incomplete penetrance. Crucially, RNA analysis of amniocytes carrying the c.1310-1G > A variant revealed only 4% aberrant splicing (c.1310del, p.Gly437Glufs*6), explaining the mild fetal phenotype of isolated femoral shortening and challenging conventional assumptions about the pathogenicity of canonical splice-site variants. The postnatal patient primarily presents with retinal pigmentary degeneration, hearing impairment, bilateral cryptorchidism, short stature, and global developmental delay. Most of these features have been reported in *WBP11*-related cases but are not considered classic diagnostic criteria for VACTERL. Notably, prenatal cases predominantly presented with cardiac malformations, whereas vertebral anomalies, common postnatally, were absent prenatally, suggesting age-dependent phenotypic evolution.

**Conclusions:**

We have documented the retinal pigment phenotype in *WBP11* mutant patients for the first time. Our findings expand the mutational and clinical spectrum of *WBP11*-related disease, highlight the limitations of sequence-based variant interpretation without functional or transcriptomic validation, and underscore the necessity of integrating detailed phenotypic correlation into prenatal genetic counseling.

**Supplementary Information:**

The online version contains supplementary material available at 10.1186/s40246-026-00966-3.

## Introduction

The WW domain-binding protein 11 (*WBP11*) gene is located on human chromosome 12p12.3 and encodes a regulatory subunit of protein phosphatase 1, a key enzyme that governs diverse cellular processes including cell division, signal transduction, and metabolic regulation [1, 2]. Mechanistically, WBP11 functions as a component of the spliceosome complex and directly activates pre-messenger RNA (pre-mRNA) splicing [3]. A canonical example is its role in regulating centriole duplication by modulating the splicing of *TUBGCP6* pre-mRNA [4]. Consequently, dysregulation of WBP11 is hypothesized to disrupt these essential cellular functions, leading to widespread functional deficits and impairing normal cellular physiology.

Until 2020, heterozygous loss-of-function variants in *WBP11* was first documented to be strongly associated with VACTERL syndrome [5], which is usually characterized by multi-system abnormalities, which can include craniofacial features, atrial septal defect, bicuspid aortic valve, pulmonic stenosis, ventricular septal defect, tracheo-oesophageal fistula, oesophageal atresia, attention deficit hyperactivity disorder, developmental delay, and intellectual disability [6]. In this study, a total of 6 *WBP11* variants were identified among 13 patients from seven families [5]. Overall, the phenotypes of these patients fall within the spectrum of multiple congenital malformations associated with VACTERL syndrome but exhibit a certain degree of heterogeneity. For instance, *WBP11-*related patients may present only with renal hypodysplasia. Additionally, microcephaly, a rare feature in VACTERL syndrome, was documented in at least two cases with *WBP11* variants [5]. Notably, a recent case report indicated that individuals affected by *WBP11* variant may also exhibit isolated vertebral anomalies or Sprengel deformity [7]. These findings suggest that the phenotypes associated with *WBP11* variants may be highly heterogeneous, and more cases need to be accumulated to fully describe their clinical feature spectrum. Moreover, the significant structural malformations associated with this disease suggest a high likelihood of prenatal detection in fetuses. However, to date, only one fetal case who had cystic brain malformation and growth restriction has been reported recently [8]. No systematic prenatal case series has been reported, and the natural history of *WBP11*-related disorder remains unclear.

Here, we reported five unrelated Chinese families with previously unreported heterozygous *WBP11* variants, including four prenatal and one postnatal cases. We conducted a comprehensive assessment of the pathogenicity of the identified variants and analyzed the characteristic prenatal and postnatal phenotypic features of *WBP11* variants in the context of previously reported cases.

## Materials and methods

### Patients

**Fig. 1:**
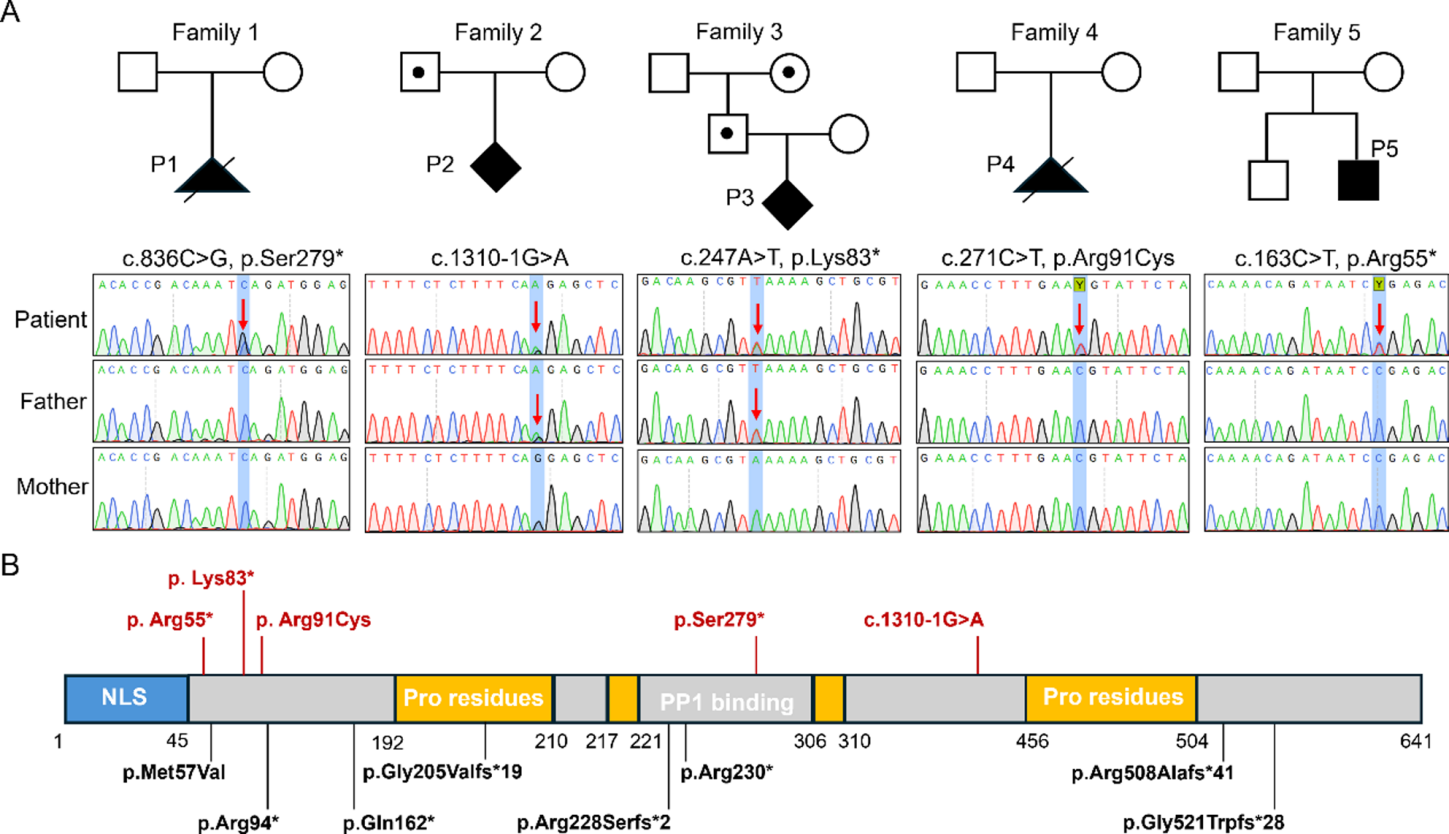
Identification and description of the *WBP11* variants. **A** Pedigrees of the patients included in this study and Sanger sequencing chromatograms of the identified *WBP11* variants. The *WBP11* variant in the fetus from Family 2 (P2) was inherited from the father, whereas the variant in the fetus from Family 3 (P3) was inherited from both the father and the paternal grandmother. All other patients harbored de novo variants. The red arrow indicates the mutated nucleotide. **B** Schematic diagram showing the distribution of all identified *WBP11* variants. Variants identified in this study are marked in red, and previously reported variants are marked in black

Four fetuses with structural malformations and one child with syndromic developmental delay, from five unrelated families (Fig. 1A), were enrolled in this study. The parents in each family were non-consanguineous.

### Genetic sequencing

All families underwent trio based whole-exome sequencing (trio-WES), which was performed as previously described [9, 10]. Briefly, the target gene exons and adjacent splice regions were captured and enriched using KAPA HyperExome probe panel (Roche, Basel, Switzerland), followed by the sequencing on the MGISEQ-2000 (BGI, Shenzhen, China) or NovaSeq 6000 (Illumina, San Diego, CA, USA) sequencing platform according to the manufacturer’s instructions. Sequencing data was assessed with average sequencing depth ≥ 200 x and ≥ 20x average depth for ≥ 98.5% of target regions. Upon alignment of the sequencing reads against the UCSC hg19 reference using Burrows-Wheeler Alignment (BWA), duplicate reads were removed. The base quality was used to recalibrate detection of SNVs, INDELs, and genotypes with GATK. Each candidate *WBP11* variant (NM_016312.3) was validated by Sanger sequencing, and the sequencing primers are available upon request.

In addition, given the urgency of the prenatal intervention window and the need to rapidly identify potential genetic etiologies, each fetus simultaneously underwent karyotyping to assess chromosomal aneuploidies, as well as either chromosomal microarray analysis (CMA, P1, P2 and P3) or copy number variation sequencing (CNV-seq) coupled with quantitative fluorescent polymerase chain reaction (P4) to evaluate CNVs.

### Amniotic fluid cell culture

Upon receipt, amniotic fluid specimens were centrifuged within 0.5 h at 1,000 × g for 10 min. The supernatant was discarded, and the remaining 1 mL cell pellet was resuspended and inoculated into culture flasks containing 4 mL of CHANG Medium D amniocyte culture medium (Fujifilm Irvine Scientific, Inc., Santa Ana, CA, USA). Cells were incubated in a humidified atmosphere at 37 °C with 5% CO₂. Medium replacement was performed after one week when dense cell colonies were observed under an inverted microscope. Cells were harvested 2–3 days following the media change, once dense colonies and a significant number of translucent round cells appeared.

### Reverse transcription PCR and TA-Cloning

Total RNA was extracted from cultured amniocytes using RNAiso Plus (TaKaRa, Dalian, China), followed by cDNA synthesis with a reverse transcription kit (TaKaRa, Dalian, China ) to create a template for splicing analysis. A PCR amplicon spanning exons 7 to 12 of the *WBP11* transcript, which encompasses the variant location in intron 10, was amplified using gene-specific primers (forward: 5’-GGTTTTGCCCTAGATCTTCCCC-3’; reverse: 5’-TGGCTGCACTTGTATCATCCG-3’). *GAPDH* served as an internal control to assess RNA quality. A 146-bp fragment spanning exons 7–8 of the *GAPDH* gene was amplified by PCR using the following primers: Forward, 5’-GTTCCTGCACCACCAACTGCTT-3’, Reverse, 5’-CCATCACGCCACAGTTTCCC-3’. The resulting PCR products were resolved on a 2% agarose gel for preliminary size analysis and then subjected to direct Sanger sequencing. For the TA-cloning assay, the PCR products were cloned into the pCE2TA/Blunt-Zero vector (Vazyme, Nanjing, China). The recombinant plasmids were transformed into competent *E. coli* DH5α cells, and positive clones were selected on LB agar plates containing ampicillin (100 µg/mL). Positive clones were identified by Sanger sequencing.

### Three-dimensional structure analysis of the WBP11 mutant protein

The crystal structure of wild-type WBP11 was retrieved from the AlphaFold Protein Structure Database (https://alphafold.ebi.ac.uk/entry/Q9Y2W2) and visualized using PyMOL software (v.1.8.4.0, Schrödinger Inc., New York, NY, USA). The p.Arg91Cys mutant was modeled using the SWISS-MODEL homology modeling server (https://swissmodel.expasy.org/). The resulting three-dimensional structures of the mutant proteins were subsequently visualized and refined in PyMOL.

## Results

### Identification of the *WBP11* variants

Five distinct heterozygous variants were identified in the *WBP11* gene (NM_016312.3) (Fig. 1A), including three nonsense variants (p.Ser279*, p.Lys83* and p.Arg55*) and one each of splice-site (c.1310-1G > A) and missense (p.Arg91Cys) variant. Except for the p.Arg91Cys variant, which was present at extremely low frequencies in the gnomAD population (v4.1.0: 0.0002%, 4 heterozygotes), the remaining four variants have not been included in any control databases (Supplementary Table 1). All variants have not been reported previously. Three of these variants (in P1, P4, P5) occurred de novo, while the variants in P2 and P3 were paternally inherited. Furthermore, karyotyping and CNV analyses yielded negative results in all four fetal cases (Supplementary Table 2). Analysis based on WES data also indicated no suspicious CNVs in case P5. Integrated analysis with eight previously reported pathogenic variants revealed that all variants were scattered across multiple functional domains of the WBP11 protein (Fig. 1B). Among the total 13 variants, 11 were loss-of-function (LOF) variants and 2 were missense variants, consistent with a haploinsufficiency disease mechanism for *WBP11*.

### Clinical features of the prenatal cases

**Fig. 2:**
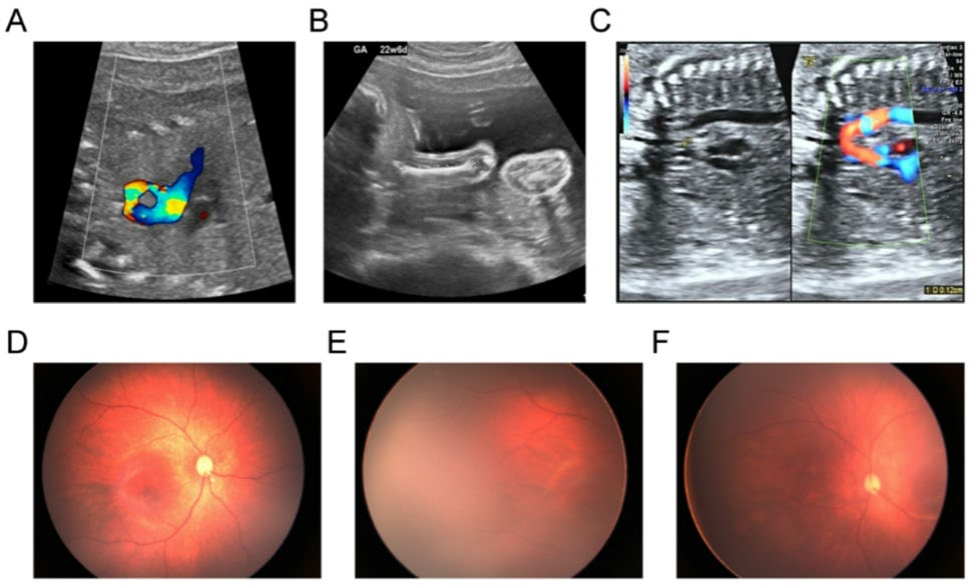
Clinical manifestations of the affected individuals. **A** Prenatal ultrasound at 23 weeks of gestation in P1 revealed pulmonary atresia with ventricular septal defect and overriding aorta. **B** At 22^+ 6^ weeks of gestation, ultrasound evaluation of P2 demonstrated femoral shortening, indicating impaired femoral development. **C** Prenatal ultrasound at 22 weeks of gestation showed P4 had aortic stenosis and ventricular septal defect. Ophthalmologic examination of Patient 5 revealed a choroidal coloboma (**D**) and punctate white lesions in the temporal retina of the right eye (**E**), and pigmentary alterations consistent with retinal dystrophy in the left eye (**F**)

Structural malformations of varying severity were detected in all four fetuses, primarily involving cardiac, renal, and skeletal malformations (Table 1 and Supplementary Table 3). P1 was referred for prenatal ultrasound at 16 weeks of gestation due to poorly visualized nasal bone. As the pregnancy progressed to 19^+ 6^weeks, a suspected cardiac anomaly was identified during a follow-up scan. Further examination at 23^+ 1^ weeks of gestation confirmed multiple cardiac anomalies, including pulmonary atresia with ventricular septal defect and overriding aorta (Fig. 2A), and central nervous system involvement manifested as microcephaly. For P2, ultrasound examination at 22⁺⁶ weeks of gestation revealed isolated bilateral femoral shortening (34 mm, 2.3rd percentile; Fig. 2B), and suspected fetal growth restriction (FGR) with abdominal circumference of 165 mm (3.2nd percentile). At follow-up at 25 weeks of gestation, the fetal femur length and abdominal circumference measured 41 mm (5.9th percentile) and 197 mm (22.1st percentile), respectively. P3 was noted to have increased nuchal translucency (3.9 mm) at 14^+ 2^ weeks of gestation. Subsequent ultrasound at 19 weeks revealed right renal agenesis, suspected FGR, suspected left duplicated kidney and an intracardiac echogenic focus. Serial follow-up ultrasounds at 24^+ 6^ and 31^+ 1^ weeks of gestation confirmed the persistence of renal anomalies. P4 was found at 21^+ 4^ weeks of gestation to have aortic stenosis and ventricular septal defect (Fig. 2C).

**Table 1:** Summary of clinical and genetic characteristics of the patients in this study

	Major clinical features	Clinical outcome	WBP11 variant (NM_016312.2)	Origin	ACMG classification
P1	A fetus at 23 weeks of gestation was found to have microcephaly, pulmonary atresia with ventricular septal defect and overriding aorta	TOP	c.836 C > G; *p*.Ser279*	De novo	*P* (PVS1+PS2_ Moderate+PM2_Supporting)
P2	A fetus at 22^+ 6^ weeks of gestation was found to have bilateral femoral shortening and suspected fetal growth restriction	Full-term delivery	c.1310-1G > A	Paternal	VUS (PVS1_Supporting+PM2_Supporting)
P3	A fetus at 19 weeks of gestation exhibited absent right kidney, suspected fetal growth restriction, possible left duplex kidney and echogenic intracardiac focus. Renal anomalies persisted on follow-up at 25 and 31 weeks.	Full-term delivery	c.247 A > T; p.Lys83*	Paternal	LP (PVS1+PM2_Supporting)
P4	A fetus at 21^+ 4^ weeks of gestation had aortic stenosis and ventricular septal defect	TOP	c.271 C > T; p.Arg91Cys	De novo	LP (PS2_ Moderate+PM1 + PP2+PP3)
P5	An 11-month-old boy mainly presenting with retinitis pigmentosa, hearing impairment, nasal septal deviation, bilateral cryptorchidism, short stature, global developmental delay, and patent foramen ovale	Survive	c.163 C > T; p.Arg55*	De novo	P (PVS1 + PS2+PM2_Supporting)

LP likely pathogenic; P pathogenic; TOP termination of pregnancy; VUS variant of uncertain significance

Notably, the fathers in Families 2 and 3 who carry *WBP11* variant exhibit suboptimal stature [165 cm (7.4th percentile) and 168 cm (14.1st percentile), respectively]. Additionally, the grandmother of P3 is also a heterozygous carrier of the p.Lys83* variant and exhibits more pronounced short stature (150 cm, 2.4th percentile). No other identified clinical abnormalities have been observed in these adult individuals based on their self-reported annual health records.

### Clinical features of the postnatal case

The proband in Family 5 was an 11-month-old male presenting with multiple congenital anomalies and global developmental delay.

During the late third trimester, the mother developed sudden, unexplained polyhydramnios that was not medically managed. At 37⁺⁵ weeks of gestation, she underwent cesarean delivery due to a uterine scar. The infant was born with a birth weight of 2400 g (0.9th percentile), length of 48 cm (9.2nd percentile), and head circumference of 32 cm (1.9th percentile), consistent with intrauterine growth restriction (IUGR). Ophthalmologic evaluation revealed persistent ocular abnormalities. Fundus examinations performed at three time points, during the neonatal period, at 4-month, and again at 9-month of age, consistently demonstrated a focal choroidal coloboma located inferior to the optic disc, accompanied by punctate white retinal lesions in the right eye (Fig. 2D and E), as well as retinitis pigmentosa in the left eye (Fig. 2F). Audiological assessment showed sensorineural hearing impairment confined to the right ear: both otoacoustic emissions and auditory brainstem response testing failed in the right ear during the neonatal period and at 4-month of age, confirming a diagnosis of unilateral sensorineural hearing loss.

The patient was initially exclusively breastfed. Weight and linear growth were adequate during the first 5 months of life, with measurements of 6.87 kg (7.7^th^ percentile) and 65.7 cm (30th percentile) at 5-month age, respectively. However, growth velocity markedly decelerated thereafter, with only minimal gain by 11-month of age, reaching just 7.2 kg (< 1st percentile) in weight and 66 cm (< 1st percentile) in height, which is indicative of postnatal growth failure. Neurodevelopmental assessment at 11-month and 18-day of age using the Gesell Developmental Scales revealed global developmental delay, with a developmental age of 8.2 months and a developmental quotient of 71. Domain-specific scores were as follows: adaptability 79, gross motor 73, fine motor 78, language 56, and social 66, with language being the most affected domain.

Additional dysmorphic features included nasal septal deviation, bilateral cryptorchidism, a patent foramen ovale (2.0 mm), mild dyscoordination of finger movements, and clinodactyly with inward curvature of the nail plate of the fourth toe on the right foot.

To rule out other genetic causes of hearing impairment with retinal involvement (e.g., Usher syndrome) [11], we performed in silico virtual panel analysis of genes associated with auditory and retinal disorders, as well as a pipeline for novel disease-causing gene discovery. No pathogenic variants other than the *WBP11* variant (p.Arg55*) were identified.

### Pathogenicity assessment of the *WBP11* variants

**Fig. 3:**
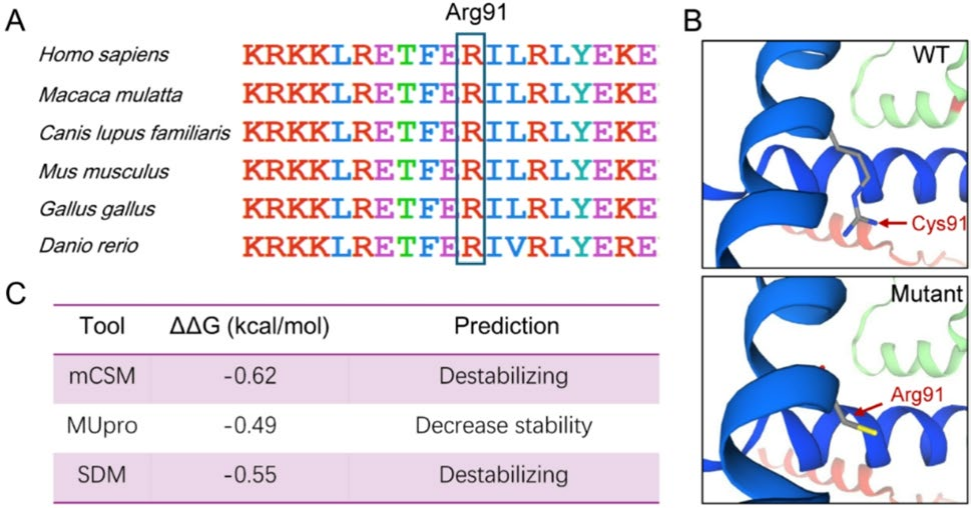
Bioinformatic analysis of the p.Arg91Cys variant. **A** The p.Arg91 residue is highly conserved across multiple species. **B** 3D structural analysis indicates that the p.Arg91Cys variant disrupts the α-helical of the WBP11 protein, respectively. **C** Protein stability of the p.Arg91Cys mutant was evaluated using multiple in silico prediction tools

Among the identified variants, the three nonsense variants (p.Ser279*, p.Lys83* and p.Arg55*) locate closer to the N-terminus of the full-length 641-amino-acid WBP11 protein (Fig. 1B). These variants were predicted to trigger nonsense-mediated mRNA decay (NMD) by AutoPVS1 [12], resulting in haploinsufficiency. According to the ACMG/AMP guidelines [13], they were classified as pathogenic or likely pathogenic (Table 1 and Supplementary Table 1). For the p.Arg91Cys variant, bioinformatic analyses indicate that this residue is highly evolutionarily conserved (Fig. 3A). The p.Arg91Cys substitution is predicted to compromise the stability of an α-helix (Fig. 3B). Moreover, in silico stability predictions using multiple online tools, including mCSM, MUpro and SDM [14–16], consistently suggest that the p.Arg91Cys variant reduces the structural stability of the WBP11 protein (Fig. 3C). Accordingly, this variant meets the criteria PS2_Moderate, PM1 [17], PP2 (z-score = 3.42 in gnomAD v4.1.0) and PP3, and was classified as likely pathogenic (Table 1 and Supplementary Table 1).

### The c.1310-1G > a variant causes incomplete splicing disruption of *WBP11* mRNA

**Fig. 4:**
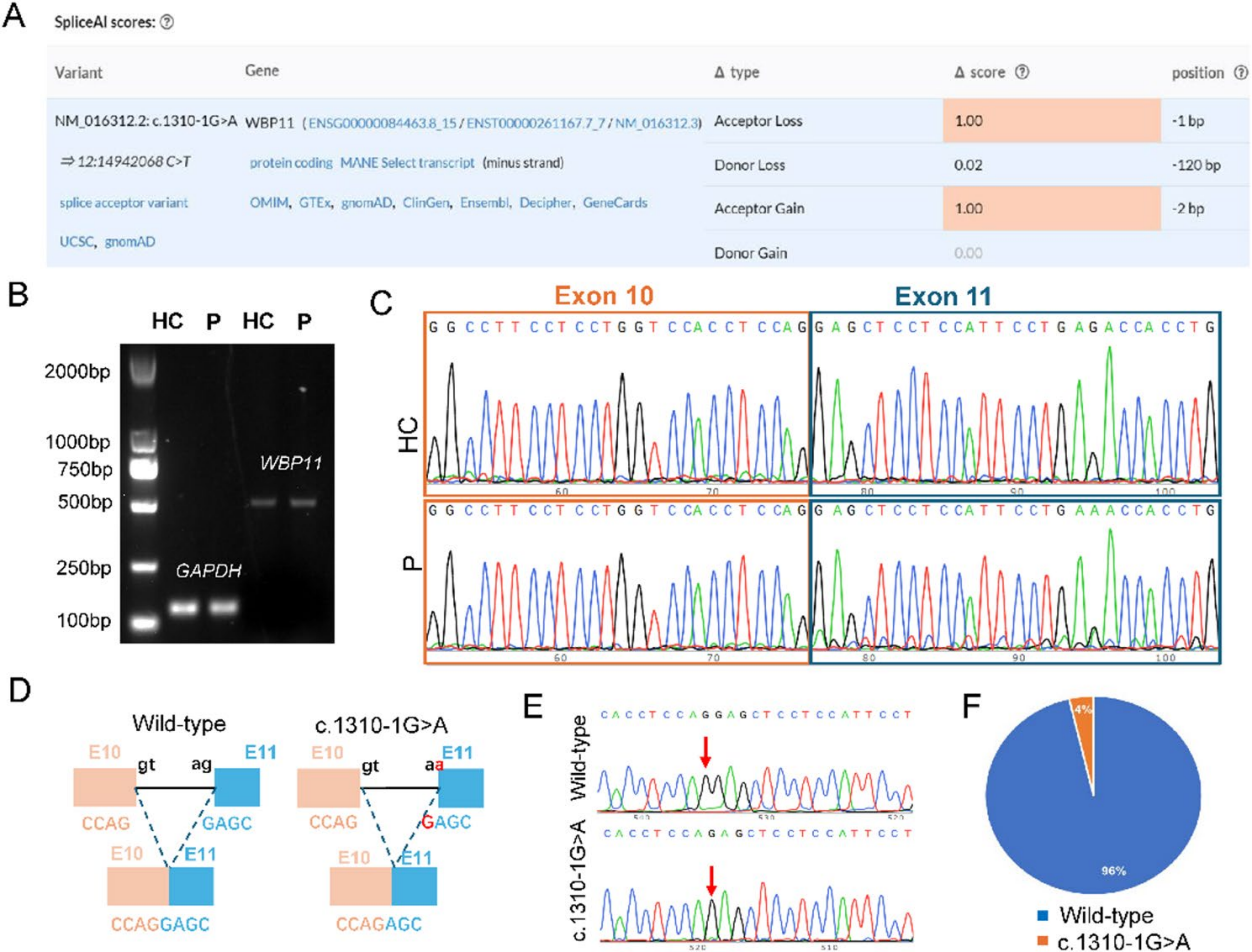
RNA analysis of the c.1310-1G > A variant. **A** SpliceAI prediction output for the c.1310-1G > A variant, indicating a high probability of splice site loss. **B** RT-PCR analysis of *WBP11* transcripts in amniotic cells from a healthy control (HC) and the patient (P). **C** Sanger sequencing of RT-PCR products from (**A**), comparing the wild-type sequence with the patient’s sequence. **D** Schematic representation of the wild-type mRNA and the predicted aberrant transcript caused by the c.1310-1G > A variant. **E** Representative Sanger sequencing results of individual TA clones derived from the patient’s RT-PCR product. **F** Quantification of TA-clone sequencing. Among 83 sequenced clones pooled from two independent experiments, 3 (4%) harbored the aberrant splicing transcript c.1310del (p.Gly437Glufs*6)

The *WBP11* gene comprises 12 coding exons spanning 1,926 base pairs. The c.1310-1G > A variant is located at the canonical splice acceptor site immediately upstream of exon 11. By SpliceAI [18] predicts that this variant completely abolishes the natural acceptor splice site and activates a cryptic acceptor site at position c.1310-2 (Fig. 4A). Consequently, splicing of the terminal two exons (exons 11 and 12) is predicted to be aberrant, resulting in a frameshift and a premature termination codon. The aberrant transcript is therefore expected to undergo NMD, leading to loss of the C-terminal region, which constitutes 32% of the full-length WBP11 protein, and likely causing loss of function. However, it remains challenging to explain the mild phenotypes observed in both the fetus and the father (Family 2). To elucidate the impact of the c.1310-1G > A variant on *WBP11* mRNA splicing, we performed RNA analysis using cultured amniocytes. Agarose gel electrophoresis of the PCR products revealed a single band that migrated to the same position in both the control and affected samples, indicating no detectable size difference (Fig. 4B). However, subsequent Sanger sequencing of the patient-derived PCR product revealed low-level mutant peaks. Sequence analysis showed that these resulted from a one-base deletion (c.1310del, p.Gly437Glufs*6), leading to a frameshift and a premature termination codon (Fig. 4C and D). Quantification of the TA-cloning results indicated that this c.1310del transcript represented only 4% of the total *WBP11* cDNA fragments analyzed (Fig. 4E and F). These results indicate that the c.1310-1G > A variant does induce aberrant splicing of WBP11 transcripts, but only at a very low frequency, suggesting minimal impact on overall WBP11 protein function and likely underlying the mild phenotypic presentation.

### Pregnancy outcome

Following genetic counseling, Families 2 and 3 chose to continue the pregnancy. P2 was born at full term and was three months old, with no apparent abnormalities observed to date. In Family 3, a male infant was born via full-term cesarean section, with no abnormalities observed in his length, weight, or head circumference (Supplementary Table 3). However, the parents declined a neonatal renal ultrasound examination. The other two families elected to undergo pregnancy termination for medical reasons but declined post-mortem X-ray and autopsy.

## Discussion

In this study, we report a Chinese case series of patients with *WBP11* variants, primarily composed of prenatal cases. We identified five heterozygous *WBP11* variants across five families, including three null variants (p.Ser279*, p.Lys83*, and p.Arg55*) classified as (likely) pathogenic, and one missense variant (p.Arg91Cys) classified as likely pathogenic. The associated clinical features of these four cases all fall within the phenotypic spectrum previously reported patients. Furthermore, we identified a paternally inherited splice-site variant, c.1310-1G > A, in a fetus presenting with isolated femoral shortening. The atypical clinical presentation prompted us to re-evaluate the pathogenicity of this splice variant using patient-derived cells. RNA analyses revealed that this variant indeed caused only a low proportion of aberrant mRNA splicing, which may account for the mild femoral developmental abnormalities observed in the fetus. Therefore, it should not be classified as a pathogenic or likely pathogenic variant within the current framework of evidence. Our findings highlight the significance of transcript validation for prenatal decision-making.

Our findings yet underscore the genetic heterogeneity and complexity associated with *WBP11* variants. First, despite being heterozygous for *WBP11* variant, the fathers of Families 2 and 3 and the paternal grandmother in Family 3 displayed no identified clinical abnormalities beyond short stature. This finding partially supports the hypothesis of incomplete penetrance in *WBP11*-related disease, which has been documented in prior report [5]. Second, although the canonical splice-site variant c.1310-1G > A is predicted to be deleterious, it does not appear to cause severe mRNA splicing defects. While the mechanism by which this variant preserves partial WBP11 function remains unclear, our data suggest that variants classified as pathogenic based on sequence-level criteria do not always result in overt clinical phenotypes. This has important implications for prenatal genetic counseling, as it cautions against pregnancy termination decisions based solely on variant identification without comprehensive phenotypic correlation.

This study also indicates the phenotypic heterogeneity resulted from *WBP11* variants. We observed marked phenotypic variability among the prenatal cases. Two fetuses (P2 and P3) exhibited suspected FGR in the first trimester; however, this finding resolved with advancing gestational age. In contrast, one fetus (P1) demonstrated progressive development of microcephaly. Additionally, the right renal agenesis initially suspected in P3 was later reclassified as ectopic kidney upon detailed evaluation in late gestation. Moreover, the only pediatric patient in this cohort did not exhibit classic features of VACTERL syndrome, though his primary manifestations, including hearing impairment, bilateral cryptorchidism, short stature, and global developmental delay, have all been reported in individuals with *WBP11* variants in previous studies. Our study also provides the first evidence that *WBP11* variants can lead to overt ocular abnormalities, including choroidal coloboma and retinitis pigmentosa, which were not reported in previously described patients.

To date, a total of 16 patients with *WBP11* variants have been reported, with only one fetal case [5, 7, 8, 19]. Summary and analysis of clinical features of the postnatal cases showed that although there is a certain degree of phenotypic variation, most patients exhibit vertebral deformities, often accompanied by craniofacial, cardiac (e.g., ventricular septal defects), or gastrointestinal abnormalities. However, prenatal cases were predominantly characterized by cardiac and central nervous system malformations. Notably, the spinal deformities, which are the most prominent feature in postnatal patients, have not been reported to date in prenatal cases (Table 2). This may be because routine prenatal ultrasound screening for fetal anomalies is typically performed at 22–23 weeks of gestation, at which point spinal abnormalities may not yet be fully apparent. That implies that individuals with *WBP11* variants who present with isolated spinal malformations may miss the opportunity for prenatal diagnosis.

**Table 2:** Comparison of clinical phenotypes between prenatal and postnatal cases with *WBP11* variants

	16 postnatal cases(1 from this study and 15 from the literature)	5 prenatal cases(4 from this study and 1 from the literature)
Vertebral deformity	8/13	0/5
Craniofacial deformity	8/14	0/5
Cardiac malformations	8/13	3/5
Gastrointestinal deformity	7/14	0/5
Sprengel deformity	6/12	0/5
Limb deformity	5/13	1/5
Renal deformity	5/13	1/5
Neurodevelopment deformity	3/14	2/5

WBP11 is known to participate in pre-mRNA splicing, however, the precise pathogenic mechanism underlying VACTERL syndrome remains incompletely understood. Emerging evidence implicates several genes, including *ZIC* and *HOXD13*, in this syndrome. Specifically, ZIC3 and its downstream target HOXD13 contribute to disease pathogenesis by regulating sonic hedgehog (SHH) and WNT signaling pathways [20–23]. Independently, WBP11 has been shown to modulate WNT signaling through its interaction with KIAA1199 [24], suggesting that *WBP11* variants may contribute to VACTERL syndrome via dysregulation of WNT signaling. Additionally, variants in the *TTLL11* gene have also been linked to VACTERL syndrome [25]. TTLL11 could regulate the primary tubulin chain elongation [26], and its functional deficiency leads to a defect at the primary cilia formation, and its functional deficiency impairs primary cilia formation [27]. It is noteworthy that WBP11 is essential for centriole development [4], which in turn is critical for the formation of primary cilia [28], a key organelles that mediate SHH and WNT signaling [29, 30]. Future work should focus on identifying which key ciliogenesis-related genes are mis-spliced due to WBP11 deficiency, thereby elucidating its molecular pathogenic mechanism.

Our study has several limitations. First, we did not further validate the pathogenicity of the missense variant through functional experiments, although multiple bioinformatic approaches consistently predicted them to be deleterious. Similarly, although the three nonsense/frameshift variants were predicted to be loss-of-function, their functional consequences remain theoretical and have not been experimentally validated in patients-derived tissues. Second, our findings do not provide new insights into the basis of phenotypic heterogeneity or disease severity, which are critical considerations for prenatal genetic counseling. Third, whether the prenatal phenotypes linked to *WBP11* variants in our cohort are representative of the broader spectrum requires verification in larger studies. Moreover, the absence of formal cognitive and imaging assessments in adult carriers, who were assessed via self-report, may limit the certainty of our conclusions regarding incomplete penetrance.

## Conclusion

In summary, we report the first prenatal case series of *WBP11* variants in Chinese patients and compare their findings with postnatal cases, laying a foundation for a comprehensive understanding of this rare disorder. Our study highlights the complexity of *WBP11*-related disease, including incomplete penetrance, marked clinical heterogeneity, and challenges in assessing the pathogenicity of splicing variant. The establishment of larger cohorts combined with rigorous basic research is essential to address these unresolved clinical questions.

## Supplementary Information

Below is the link to the electronic supplementary material.

Supplementary Material 1.

Supplementary Material 2.

Supplementary Material 3.

## Data Availability

The raw data of WES for this study is not publicly available because of privacy concerns from the families. The data can be obtained from the corresponding author Niu Li ( liniu0509@163.com ) by researchers who fulfill the requirements for accessing confidential data.

## Abbreviations

pre-mRNA: Pre-messenger RNA
WES: Whole-exome sequencing
LOF: Loss-of-function
SD: Standard deviation
