# Supplementary material for: Prenatal and postnatal manifestations of WBP11-related disorder in Chinese patients: expanding the phenotypic and mutational spectrum
Source: Hum Genomics. 2026 Apr 13;20:90. doi: 10.1186/s40246-026-00966-3 (PMC13200368; doi:10.1186/s40246-026-00966-3)
Supplement: Supplementary file 1 — Supplementary Material 1. [file 40246_2026_966_MOESM1_ESM.docx]

Supplementary Table 1. Detail information of the *WBP11* variants reported in this study

| *WBP11* variant | | | AF in gnomAD (v4.1.0) | ACMG criteria |
| --- | --- | --- | --- | --- |
| cDNA level (NM_016312.2, a total of 12 exons) | Protein level (NP_057396.1, a total of 641 amino acids) | Genomic coordinate (GRCh 37) |  |  |
| c.836C>G (Exon 8) | p.Ser279* | chr12:14946742 G>C | Absent | Pathogenic  (1)PVS1: Predicted to undergo NMD  (2)PS2_ Moderate: De novo, downgraded due to insufficient phenotype specificity  (3)PM2_Supporting: AF=0 |
| c.1310-1G>A (Intron 10) | Not applicable | chr12:14942068 C>T | Absent | Uncertain significance  (1)PVS1_Supporting: Aberrant splicing occurs at a low frequency attributable to this variant.  (2)PM2_Supporting: AF=0 |
| c.247A>T (Exon 5) | p.Lys83* | chr12:14949881 T>A | Absent | Likely pathogenic  (1)PVS1: Predicted to undergo NMD  (2)PM2_Supporting: AF=0 |
| c.271C>T (Exon 5) | p.Arg91Cys | chr12:14949857 G>A | 0.0002%, 4 heterozygotes in gnomAD | Likely pathogenic  (1)PS2_ Moderate: De novo, downgraded due to insufficient phenotype specificity.  (2)PM1: Reduce protein stability  (3)PP2: Z score of missense=3.42  (4)PP3: Revel value=0.816; CADD_Phred value=25.1; PolyPhen2_HVAR value=0.996 |
| c.163C>T  (Exon 4) | p.Arg55* | chr12:14952596 G>A | Absent | Pathogenic  (1)PVS1: Predicted to undergo NMD  (2)PS2: De novo  (3)PM2_Supporting: AF=0 |

AF, Allele frequency; NMD: nonsense-mediated decay
