# Supplementary material for: Prenatal and postnatal manifestations of WBP11-related disorder in Chinese patients: expanding the phenotypic and mutational spectrum
Source: Hum Genomics. 2026 Apr 13;20:90. doi: 10.1186/s40246-026-00966-3 (PMC13200368; doi:10.1186/s40246-026-00966-3)
Supplement: Supplementary file 2 — Supplementary Material 2. [file 40246_2026_966_MOESM2_ESM.docx]

Supplementary Table 2. Overview of prenatal genetic testing results for four fetal cases

| Patient | Gestational age at the time of testing | Testing item | Testing results |
| --- | --- | --- | --- |
| P1 | 16^+6^ weeks | NIPT | Negative |
|  | 23^+1^ weeks | Trio-WES（BGI platform） | *WBP11* variant |
|  |  | CMA (Affymetrix CytoScan 750K） | Negative |
|  |  | Karyotyping | Negative |
| P2 | 12 weeks | NIPT | Negative |
|  | 22^+6^ weeks | Trio-WES（BGI platform） | *WBP11* variant |
|  |  | CMA (Affymetrix CytoScan 750K） | Negative |
|  |  | Karyotyping | Negative |
| P3 | 12 weeks | NIPT | Negative |
|  | 19 weeks | Trio-WES（BGI platform） | *WBP11* variant |
|  |  | CMA (Affymetrix CytoScan 750K） | Negative |
|  |  | Karyotyping | Negative |
| P4 | 15 weeks | NIPT | Negative |
|  | 21^+4^ weeks | Trio-WES（Illumina platform） | *WBP11* variant |
|  |  | CNV-seq Plus QF-PCR | Negative |
|  |  | Karyotyping | Negative |

CMA, chromosomal microarray analysis; CNV-seq, copy number variation sequencing; NIPT, non-invasive prenatal testing; QF-PCR, quantitative fluorescent polymerase chain reaction; WES, whole-exome sequencing
