# Supplementary material for: Prenatal and postnatal manifestations of WBP11-related disorder in Chinese patients: expanding the phenotypic and mutational spectrum
Source: Hum Genomics. 2026 Apr 13;20:90. doi: 10.1186/s40246-026-00966-3 (PMC13200368; doi:10.1186/s40246-026-00966-3)
Supplement: Supplementary file 3 — Supplementary Material 3. [file 40246_2026_966_MOESM3_ESM.docx]

Supplementary Table 3. Detailed clinical information for four fetal cases organized by examination timeline

| Patient | Gestational age | Ultrasound findings and measurement values (percentiles) of important indicators | Clinical conclusions |
| --- | --- | --- | --- |
| P1:  Father: 34-year old, 188 cm (94.5^th^ percentile)  Mother: 28-year old, 160cm (32.8^th^ percentile) | 12 weeks | NT 2.0mm | Normal |
|  | 16^+6^ weeks | Poorly visualized nasal bone；BPD 37 mm (58.8^th^); HC 132 mm (39.9^th^); AC 116mm (67.4^th^); FL 20 mm (17.4^th^) | Poorly visualized nasal bone |
|  | 19^+6^ weeks | (1)Ventricular septal discontinuity with a single overriding great artery; (2) Absent pulmonary valve motion; BPD 45 mm (40.6^th^); HC 168 mm (44.8^th^); AC 141mm (19.7^th^)；FL 30 mm (40.2^nd^) | Abnormal fetal heart development |
|  | 23^+1^ weeks | (1) Ventricular septal defect (3.3 mm) with aortic overriding; (2) Poor visualization of the main pulmonary artery and its branches; BPD 54 mm (26.3^rd^); HC 188 mm (0.7^th^); AC 174mm (12.2^nd^); FL 36 mm (7.6^th^) | Pulmonary atresia with ventricular septal defect and overriding aorta; Microcephaly |
|  | 23^+4^ weeks |  | TOP without autopsy |
| P2  Father: 28-year old, 165 cm (7.4^th^ percentile);  Mother: 27-year old, 150cm (2.4^th^ percentile) | 12 weeks | NT 1.6mm | Normal |
|  | 22^+6^ weeks | BPD 57 mm (73.3^rd^); HC 193 mm (8.5^th^); AC 165mm (3.2^nd^); FL 34 mm (2.3^rd^) | Bilateral femoral shortening；suspected FGR |
|  | 25 weeks | BPD 62 mm (53.7^th^); HC 227 mm (43.9^th^); AC 197mm (22.1^st^); FL 41 mm (5.9^th^) |  |
| P3  Father: 33-year old, 168 cm (14.1^st^ percentile);  Mother: 27-year old;  Grandmother: 150cm (2.4^th^ percentile) | 13^＋5^ weeks | NT 3.9mm | Abnormal |
|  | 19 weeks | (1) Inadequate visualization of the right kidney; (2) Left kidney measuring 17 × 9 mm with suspected renal pelvis duplication; (3) Right adrenal gland appearing flattened along the paraspinal region; (4) An echogenic intracardiac focus in the left ventricle; BPD 43mm (25.7^th^); HC 162mm (31.7^th^); AC 131 mm (4.1^st^); FL 28mm (22.2^nd^) | Dysplasia or absence of Right kidney, suspected left duplicated kidney; intracardiac echogenic focus; FGR possible |
|  | 24^＋6^ weeks | (1) Left kidney measuring 38 × 18 mm; (2) Suggestive of duplicated renal pelvis and dual renal arteries; (3) No definite renal parenchyma is visualized in the right renal fossa; (4) The right adrenal gland is observed lying flat adjacent to the paravertebral region; BPD 62mm (59^th^); HC 231mm (69.2^nd^); AC 205 mm (56.1^st^); FL 45mm (67^th^) | Dysplasia or absence of Right kidney; suspected left duplicated kidney |
|  | 31^＋1^ weeks | (1) Left kidney measuring 43 × 19 mm; (2) Suggestive of duplicated renal pelvis and dual renal arteries; (3) No definite renal parenchyma is visualized in the right renal fossa; (4) The right adrenal gland is observed lying flat adjacent to the paravertebral region; (5)An echogenic structure consistent with renal parenchyma is visualized in the right portion of the pelvic cavity, measuring approximately 27 × 12 mm; BPD 81mm (72.1^st^); HC 295mm (76.9^th^); AC 267 mm (42.9^th^); FL 58mm (52.9^th^) | Right pelvic ectopic kidney; suspected left duplicated kidney |
|  | 39 weeks | A male neonate was born by Cesarean delivery, with birth parameters: weight 3.15 kg (37.5^th^ percentile), length 48 cm (~16^th^ percentile), HC 34 cm (~36^th^ percentile). Renal phenotype could not be assessed because the parents declined a neonatal ultrasound examination. | No other abnormalities were detected on initial clinical examination. |
| P4  Mother: 24-year-5-month old | 11^+6^ weeks | NT 1.4mm | Normal |
|  | 21^＋4^ weeks | (1) An echogenic discontinuity (~2.7 mm) in the superior ventricular septum; (2) Pulmonary artery diameter 6.4 mm; (3) Aortic valve annulus 2.3 mm; (4) Ascending aorta 2.5 mm; (5) Aortic arch 1.0 mm; (6) Superior vena cava 2.4 mm; (7) Right atrium 7.6 mm，right ventricle 6.1mm，left atrium 5.9mm，left ventricle 6.0mm | Aortic stenosis; ventricular septal defect |
|  | 26 weeks |  | TOP without autopsy |

AC, abdominal circumference; BPD, biparietal diameter; FGR, fetal growth restriction; FL, femur length; HC, head circumference; NT, nuchal translucency; TOP, termination of pregnancy
